# Supplementary material for: Novel Mixed Cancer-Cell Models Designed to Capture Inter-Patient Tumor Heterogeneity for Accurate Evaluation of Drug Combinations
Source: Int J Mol Sci. 2025 Dec 30;27(1):413. doi: 10.3390/ijms27010413 (PMC12785625; doi:10.3390/ijms27010413)
Supplement: Supplementary file 1 [file ijms-27-00413-s001.zip › ijms-3971284-supplementary.pdf]

Supporting Information

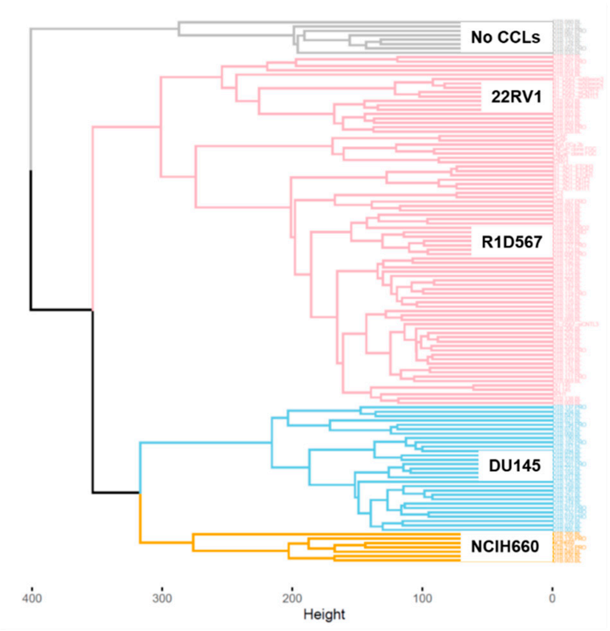

Figure S1. Hierarchical clustering of transcriptomes of SU2C/PC-WC patient tumors and 10 prostate cancer cell lines and their derivatives. A representative cell line from each cluster is highlighted in the boxes.

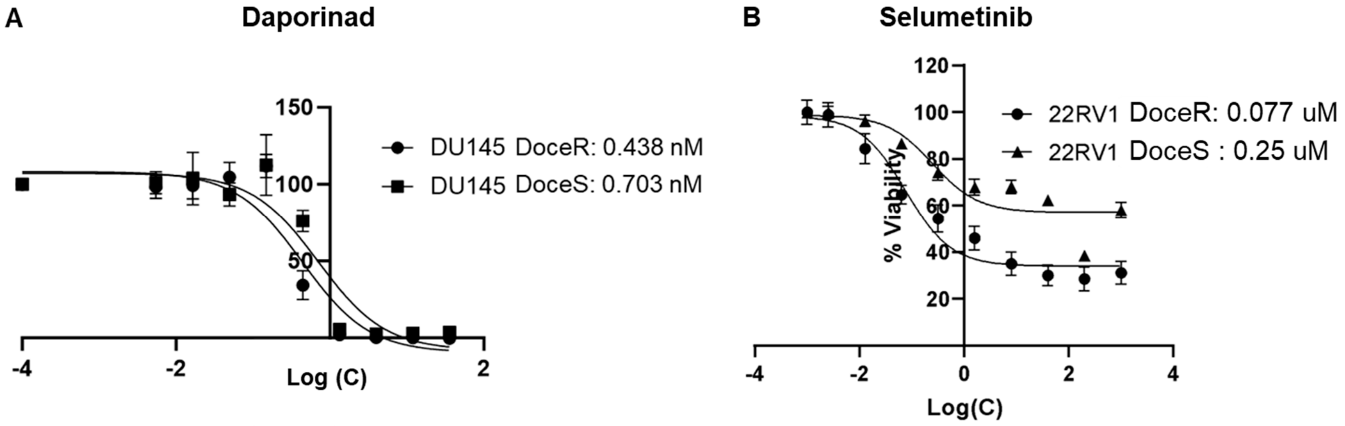

Figure S2. Dose-response curves constructed from WST absorbance data for daporinad (A) and selumetinib (B) in docetaxel resistant and sensitive clones of DU145 and 22RV1, respectively.

Table S1. IC50 values in uM of drugs screened individually in parent and docetaxel resistant clones of PCa lines 22RV1, DU145 and R1D567.

	22RV1_Doce resistant	22RV1_Parent	DU145_Doce resistant	DU145_Parent	R1D567_Doce resistant	R1D567_Parent
**Vinorelbine**	0.1	0.008569	0.02702	0.013451	0.4626	0.009241
**Daporinad**	0.008874	0.008229	0.003074	0.004229	0.004594	0.004933
**Dasatinib**	NA	2.855	NA	9.084	NA	0.4092
**Selumetinib**	0.07744	0.2527	8.175	150	54.13	10.37
**Docetaxel**	>0.7	0.0024	>0.1	0.0086	>0.1	0.0032

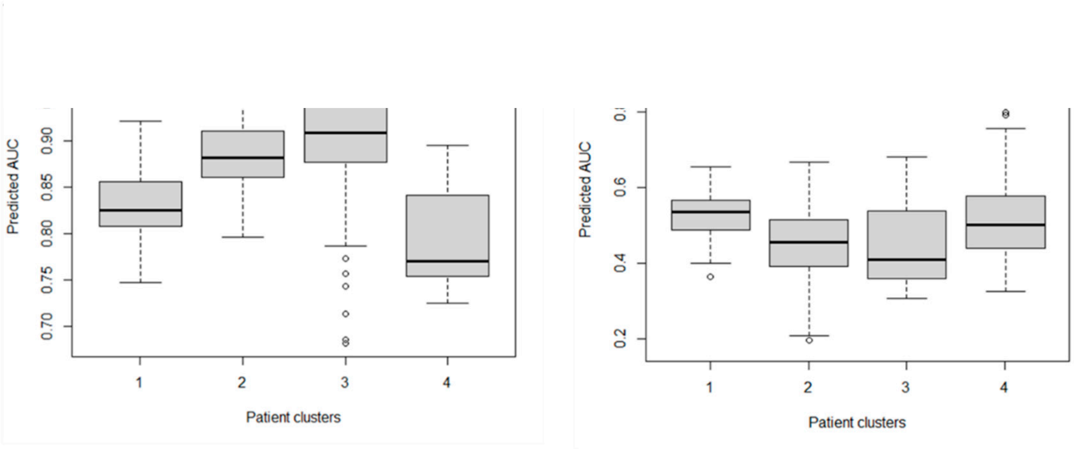

Figure S3. Oncopredict imputed drug sensitivities of SU2C-EC patients to docetaxel (A) and daporinad (B), stratified by cluster assignment obtained from hierarchical clustering of patient transcriptomes with PC lines.

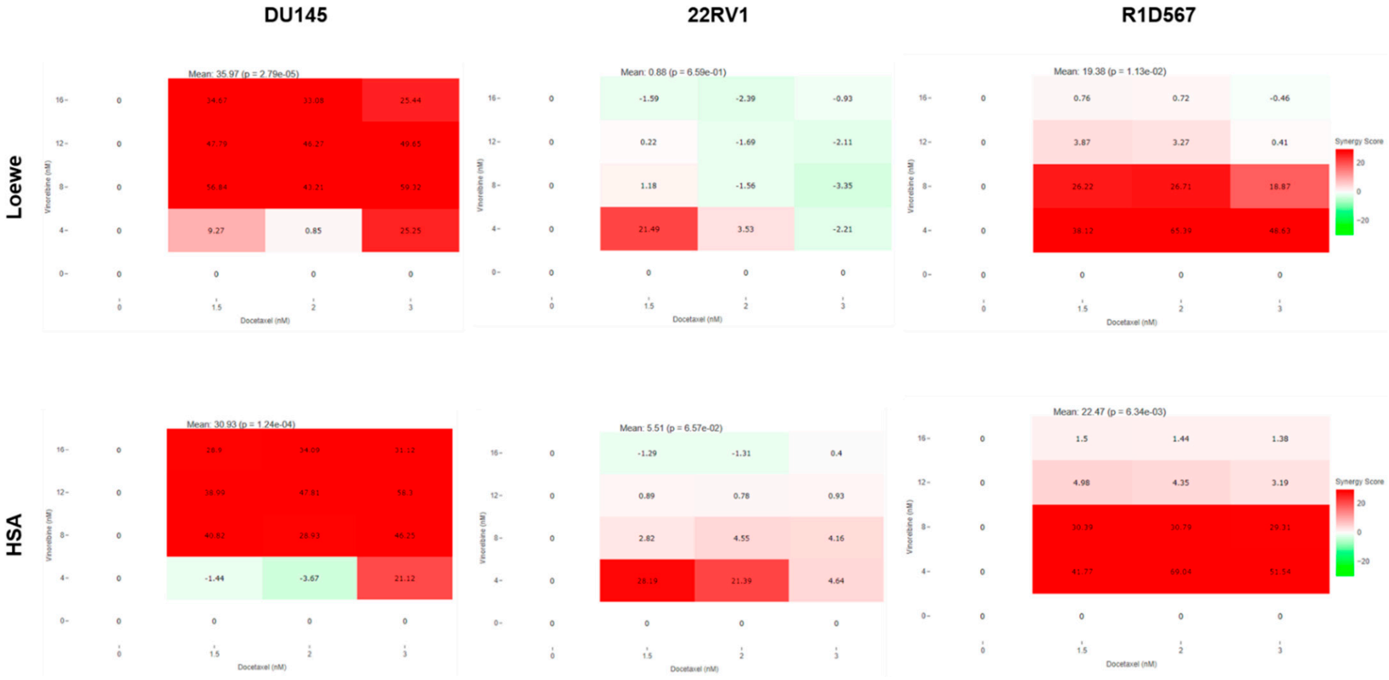

Figure S4. Synergy scores for docetaxel and vinorelbine calculated using the Loewe additivity model and Highest Single Agent (HSA) models from combination and monotherapy responses in individual cell-lines.
